# Commentary: The reliability of telomere length measurements

**DOI:** 10.1093/ije/dyv166

**Published:** 2015-09-24

**Authors:** Simon Verhulst, Ezra Susser, Pam R Factor-Litvak, Mirre JP Simons, Athanase Benetos, Troels Steenstrup, Jeremy D Kark, Abraham Aviv

**Affiliations:** ^1^Groningen Institute for Evolutionary Life Sciences, University of Groningen, Groningen, The Netherlands,; ^2^Imprints Center for Genetic and Environmental Lifecourse Studies, Department of Epidemiology, Columbia University Mailman School of Public Health, and New York State Psychiatric Institute, New York, NY, USA,; ^3^Department of Epidemiology, Columbia University Mailman School of Public Health, New York, NY, USA,; ^4^Department of Animal and Plant Sciences, University of Sheffield, Sheffield, UK,; ^5^Département de Médecine Gériatrique, and INSERM, U1116, Université de Lorraine, Vandoeuvre-les-Nancy, France,; ^6^Danske Bank, Copenhagen, Denmark,; ^7^Hebrew University-Hadassah School of Public Health and Community Medicine, Jerusalem, Israel and; ^8^Center of Human Development and Aging, Rutgers, State University of New Jersey, Newark, NJ, USA

The importance of telomere biology in human disease is increasingly recognized and, in parallel, use of telomere length (TL) measures is proliferating in epidemiological and clinical studies. Such studies measure leukocyte TL (LTL) using several methodological approaches. Shorter LTL is associated with atherosclerosis^1^ and all-cause mortality.^2^ Given the increasingly recognized role of TL in human ageing and its related diseases, it is essential to know more about the reliability and validity of TL measurement methods, their comparability and which method is optimal for a specific epidemiological/clinical setting.

In an effort to address this knowledge gap, Martin-Ruiz *et al*. (MR)^3^ studied the reliability of TL measurement techniques. They compared the popular qPCR method with the labour-intensive Southern blots (SBs) and single telomere length analysis (STELA). MR concluded that ‘neither technique nor laboratory had strong influence on result variation’, and that ‘Southern blotting and qPCR are similar in their reproducibility’. Unfortunately, for the following reasons we believe that for epidemiological studies neither conclusion is justified by the data.

## 

### Reliability of LTL

Most DNA samples (10/12) used by MR were obtained from human placenta, cell cultures and cancer cells. However, the inter-assay reliability of LTL is the pertinent parameter for epidemiological studies. MR included only two DNA samples from leukocytes and, because these were added in the second round of the study, they could not be used to measure inter-assay reliability of LTL. TL results for human placenta, cultured and cancer cells cannot be automatically generalized to LTL reliability, which is the primary concern of epidemiologists. Note also that MR used pooled leukocyte samples of multiple donors, and effects of pooling on assay reliability can therefore not be excluded. A previous comparison of LTL reliability has been done for the SB and the qPCR methods in a study^4^ cited by MR. The study reported a clear difference in inter-assay coefficient of variation (CV) between SB = 1.74% and qPCR = 6.54%, using 50 leukocyte DNA samples from individual donors. Moreover, Steenstrup *et al*.^5^ investigated whether LTL elongation in longitudinal studies can be attributed to measurement error vs a real biological phenomenon. They found little evidence for LTL elongation over and above the effects expected from measurement error. At the same time, the available data indicated a substantially larger proportion of individuals with an apparent LTL elongation in qPCR-based studies when compared with SB-based studies. In our view, the most parsimonious explanation for this finding is the higher measurement error of the qPCR method.

MR observed that rank correlations between measurements obtained in different laboratories and with different methods were high, reflecting similar rank orders of the observations. Due to the inclusion of different cell types, the range of TLs in this study (4.7-9.2 kb) is much higher, however, than the age group-specific range (about 3 kb by direct SBs within age groups) used in most epidemiological studies of LTL. This will have inflated the rank correlation beyond what is relevant for LTL in epidemiological studies considerably, contributing to the erroneous conclusion that the SB and qPCR methods yielded similar results.

### Sample size and composition

MR used 12 samples. These were measured by two laboratories using SBs, one laboratory using STELA and seven laboratories using qPCR. As both the number of samples and the number of laboratories using techniques other than qPCR were low, the statistical tests used by MR to infer no difference in reliability between methods are underpowered and consequently of limited value. We are thus left puzzled by the authors’ claim of > 95% power to detect the difference previously reported between inter-assay CVs for LTL using SBs and qPCR in 50 leukocyte DNA samples.^4^ MR provide no details of their calculation in support of this statement, nor on the exact difference between inter-assay CVs for which they calculated their statistical power.

Furthermore, the authors combined the two SB and one STELA laboratories for comparisons of inter-laboratory CV across methods. We see little scientific justification for this choice, which in effect leaves one with no information specific to either the SB or STELA technique. For the two leukocyte samples, the inter-laboratory CVs were 6.2% and 6.5% for the SB/STELA laboratories vs 22.2% and 22.2% for the qPCR laboratories (samples K and L, Table 2, in erratum MR)^6^. These results, albeit from a tiny sample size, are consistent with higher measurement error of the qPCR over SB/STELA based-methods. This is not specific for the leukocyte samples; overall the inter-laboratory CVs were substantially higher when using qPCR (*P* = 0.001 according to MR). Finally, for the crucial analyses of the inter-assay and intra-assay CVs, the total number of DNA samples was restricted to 5 and 3, respectively, and none of these were from leukocytes.

### CV as a measure of reliability

**Figure 1.**
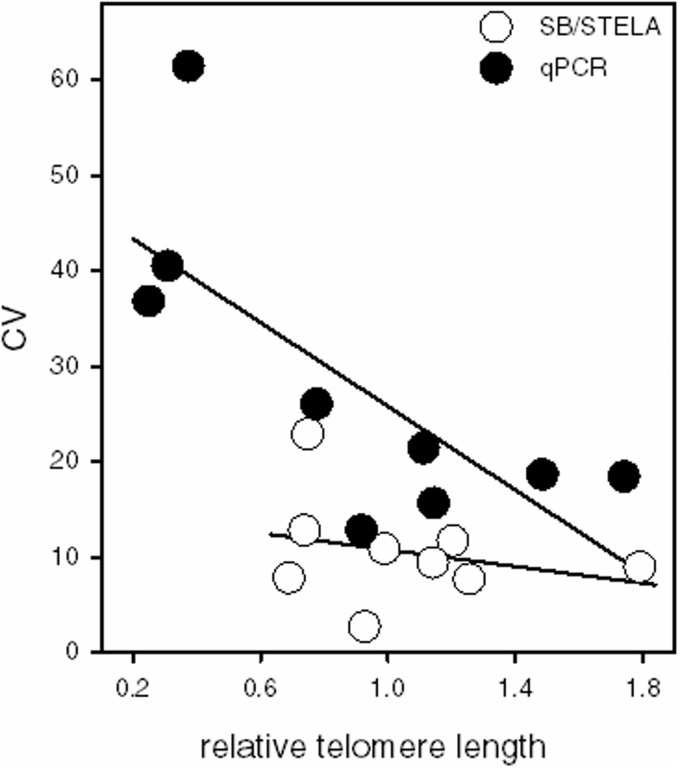
Coefficient of variation (CV%) between laboratories for SB/STELA vs qPCR plotted against telomere length. Telomere length was standardized per laboratory, dividing the results for all samples by the value obtained for sample G. The X-axis displays the average relative telomere length per sample per technique. Data from Table 2, round 1, in MR (SB/STELA R^2 ^= 0.06, qPCR R^2 ^= 0.54). Round 2 yielded similar results, except that the non-significant trend for SB/STELA was positive instead of negative.

A characteristic of the CV is its dependence on the mean, and hence the implicit assumption when using the CV is heteroscedasticity, i.e. that the variance is proportional to the mean. We examined whether this assumption holds in the results presented by MR. Figure 1 suggests that it holds for SB. There is a negligible correlation between mean and CV, which is not surprising given the logarithmic nature of molecular size ladders on gels.^7^ By contrast, Figure 1 suggests that it does not hold for qPCR. There is a strong negative correlation between average TL and CV, which implies that the error made in qPCR-based TL measurements is not proportional to the mean, but instead is closer to a constant (assay-specific) value. Such a finding undermines the CV as a reliability measure for qPCR-based TL studies. Instead we recommend using the intra-class correlation coefficient, which yields an informative estimate, provided that the ‘test’ samples are similarly distributed as the samples in the investigated population.

Figure 1 also illustrates the larger range of values obtained with qPCR when compared with SB. MR suggest that the larger ‘dynamic range’ obtained with qPCR compensates for the lower precision of the method. However, when CV values are calculated for SB laboratories alone (i.e. ignoring the STELA results), the inter-laboratory CV is in fact over 40% higher for the qPCR laboratories (paired t-test, t = 2.39, df = 18, *P* < 0.025). Therefore, the larger range in TL values obtained using qPCR compared with SBs was more likely to be caused by a lower precision of qPCR, rather than compensating for it.

### DNA quality

MR reported that they assessed DNA quality (purity and integrity) by ‘UV spectroscopy and agarose gel electrophoresis’, which is not typical of epidemiological studies that use the qPCR-based method. This may be critical if qPCR-based results are influenced by DNA integrity, which cannot be ruled out, as intact amplifiable target sequences are essential for reliable and valid results.^8^ Therefore, it is important to demonstrate in impartial studies that DNA integrity does not affect the T/S ratio results.

### Conclusions

We see little evidence in MR that the reliabilities of SB and qPCR in measuring TL are equivalent. The number of laboratories performing SBs and STELA in their study was very small, as was the number of samples examined. Furthermore, only two of the 12 samples were from human leukocytes, the standard cell type used in epidemiological studies, and the inter-assay reliability of LTL was not measured.

**Figure 2.**
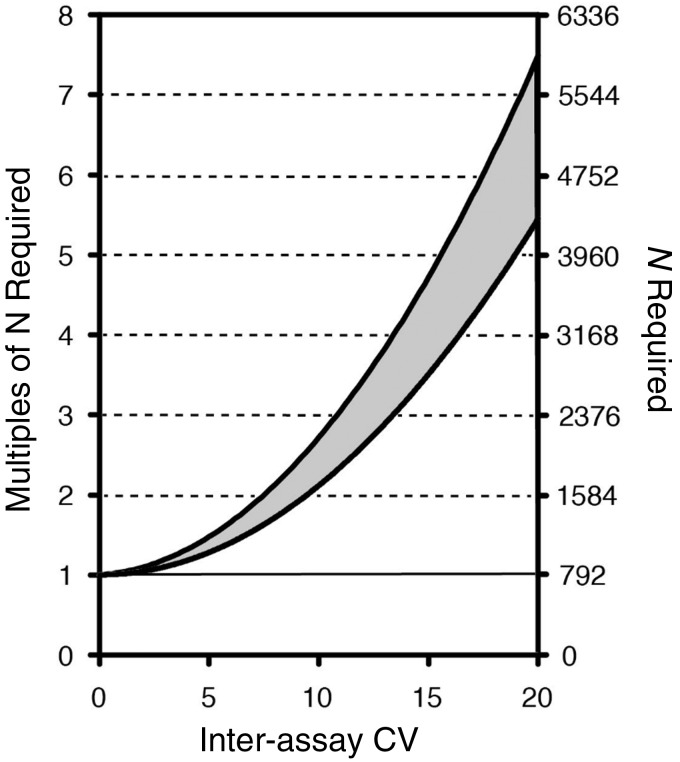
Effect of inter-assay coefficient of variation (CV%) on sample size required for a statistical power of 0.9. Shown on the left axis are the multiples of the sample size needed compared with CV = 0% (i.e. perfect reliability). The required number of multiples is independent of effect size. Shown on the right axis is the *N* required for the specific case of demonstrating a difference of 0.15 kb (approximate gender effect) with power 0.9. Calculations are based on a two-sample t-test and power analyses were carried out using G-Power, assuming an LTL average ± SD of 6.9 ± 0.65 kb. Estimates depend on the sample size used to calculate the CV due to the downward bias in SD estimates, and this bias decreases rapidly with sample size over which each CV is calculated. Upper line: CV based on sample standard deviation estimated from duplicate measurements (maximum bias). Lower line: CV based on population SD, i.e. unbiased.

The qPCR does have the advantage over SB and other methods in that it costs less and requires fewer resources, but at the expense of measurement reliability. This implies that to demonstrate the same effect statistically, a larger sample size is needed when using qPCR in comparison with using SB/STELA. It is informative therefore to examine the consequences of lower reliability (higher CVs) for the actual sample sizes required. The following example might serve to contextualize the impact of inter-assay CVs on required sample sizes. On average, women’s LTL is longer by 0.15 kb than men’s LTL. As shown in Figure 2, to detect this difference with 90% power, with an increase in inter-assay CV from 2 to 20%, the required sample size increases by approximately six-fold.

The paper by MR and this commentary highlight an issue that is of great importance to the future of telomere epidemiology. As proposed in the pages of this journal 5 years ago,^9^ large-scale epidemiological studies, based on measurements of LTL using both SB and qPCR in laboratories experienced in these techniques, are urgently needed to resolve matters related to ‘noise’ and to assess how the two methods compare in capturing the associations of LTL with a host of human traits. Without such comparison, we fear that the claim by MR that SB and qPCR are equally reliable methods to measure LTL may result in suboptimal choices of methods, thereby wasting precious resources.

**Conflict of interest:** None declared.
